# The impact of graphene oxide on the magnetic and hyperthermia properties of CoFe_2_O_4_ and MnFe_2_O_4_ ferrites

**DOI:** 10.1038/s41598-026-51345-w

**Published:** 2026-05-12

**Authors:** Wegdan Ramadan, Aya Gasser, Abdallah Ramadan, Marcos Garcia, Yohannes Getahun, Ahmed A. El-Gendy

**Affiliations:** 1 Department of Physics, Faculty of Science, Alexandria University, Alexandria, 21511 Egypt; 2 Department of Physics, University of Texas at El Paso, El Paso, TX 79968 USA

**Keywords:** Chemistry, Materials science, Nanoscience and technology, Physics

## Abstract

MnFe_2_O_4_ and CoFe_2_O_4_ nanoparticles, along with their graphene oxide (GO) composites, were synthesized and characterized for potential use in magnetic hyperthermia applications. MnFe_2_O_4_ and CoFe_2_O_4_ were synthesized through hydrothermal method and characterized using XRD, TEM, Raman, FTIR spectroscopy and VSM to determine their structural, morphological, and magnetic properties. Magnetic measurements revealed superparamagnetic behavior in MnFe_2_O_4_, while CoFe_2_O_4_ exhibited ferromagnetic properties. MnFe_2_O_4_, with soft ferrite characteristics, demonstrated higher magnetization and heating efficiency compared to CoFe_2_O_4_, a hard ferrite with higher magnetic anisotropy. The incorporation of graphene oxide introduced competing effects: while it improved colloidal dispersion and offered a surface platform amenable to bioconjugation and functionalization for targeted drug delivery, it consistently reduced SAR values across both ferrite systems. This reduction is attributed to magnetic phase dilution by the nonmagnetic GO matrix, as well as domain pinning caused by interfacial strain and structural defects, reflecting perturbations to the magnetic microstructure. Enhancing the hyperthermia performance of ferrites may require optimizing more than just saturation magnetization; factors such as the anisotropy constant of the ferrite must be tuned to match the relaxation timescale with the applied field frequency. Hyperthermia experiment demonstrated enhanced heating efficiency (SAR values) for MnFe_2_O_4_, achieving 110 W/g, compared to its GO composite (60 W/g), highlighting the synergistic effects of both Néel and Brownian relaxation mechanisms whose effective relaxation time is better matched to the applied frequency. On the other hand, CoFe_2_O_4_ system operates predominately through Brownian relaxation, as its Néel relaxation is thermally inaccessible under these conditions, resulting in a much lower SAR of 70 W/g. The composite, CoFe_2_O_4_-GO, exhibited lower but comparable SAR values, decreasing from 70 to 60 W/g, consistent with a Brownian-dominated mechanism relatively insensitive to GO-induced magnetic modifications. In this study, we identify some key parameters influencing hyperthermia performance (e.g., particle size, morphology, and magnetic anisotropy) in an effort to present optimized nanoparticles for effective hyperthermia-based cancer treatment.

## Introduction

Nowadays, researchers show more attention and interest in hyperthermia as a therapeutic method that generates suitable heat and is a promising method for cancer treatment^1^. Hyperthermia initiates a localized increase in the order of 5°–7° more than the physiological temperature, i.e. in the range of 41–45 °C. Effective local or regional hyperthermic cancer therapy can be induced by either longer heating durations at temperatures of 41–45 °C or by short duration treatment of cancer cells with higher temperatures, or both^2^. However, hyperthermia alone is not enough to be used as a sole treatment technique for cancer, but rather in a synergistic manner with other techniques like chemotherapy and radiotherapy^3,4^. According to the National Cancer Institute (NCI), there are many ways to induce localized hyperthermia like microwaves, radiofrequency, ultrasound and even light. Traditional hyperthermia techniques using radiofrequency and microwaves have their own limitations; they are invasive and lead to serious side effects. Light is another method of hyperthermia such as it causes photothermal treatment using infra-red radiation, generating heat and inducing the necrosis or apoptosis of cancer cells. Another crucial disadvantage of light is the lack of depth in its penetration into the tissues, thus proving to be futile for deep tumor therapy. Gold nanoparticles in its different shapes, carbon nano tubes and polymers such as polyaniline^5–9^ can be used for photothermal cancer therapy as they can be injected and reach the local tumor area in a targeted manner and can be stimulated via near infra-red laser light to generate heat. Oxides, including magnetic oxides/ferrites, have been used for many different electronic applications and for energy storing^10–14^. On the other hand, magnetic nanoparticles such as ferrites, iron oxide^15,16^ and superparamagnetic iron oxide nanoparticles (SPIONs)^17^ can be stimulated via alternating magnetic fields or microwaves to generate heat. Such magnetic nanoparticles can generate hyperthermia and increase the surrounding temperature to 41–45 °C. The mechanism of heat dissipation using MNPs involves a delay in the relaxation of the magnetic moments, either due to rotation within the particle (Néel) or due to rotation of the particle itself (Brownian) when an AC magnetic field is applied, in which the magnetic field reversal time is shorter than the magnetic moment relaxation of the nanoparticles^18,19^. When applying an alternating magnetic field (AMF) to magnetic nanoparticles, MNPs, the specific area of the magnetic hysteresis loop, *A*, corresponds to the dissipated energy used. The power generated by the MNPs known as specific absorption rate (SAR) and the hysteresis loop area, *A*, are linked by the equation of **SAR =** ***Af***, where *f* is the frequency of the applied alternating magnetic field^20,21^. The challenge here is the development of MNPs with high SAR performance to reduce the in vivo ferrofluid dose and minimize the potential side effects that could arise during treatment^22–24^. For medical reasons, humans cannot be exposed to an alternating magnetic field having large intensity nor having large frequency^25^. Typical reported values used for medical purposes are around 100 kHz and 20 mT^26^. Hence, it would be more convenient to resolve the optimization of other important parameters that influence SAR values like the particle size, saturation magnetization, morphology and magnetic anisotropy^27^. Such an optimization process needs a careful analysis of the combined influence of the mentioned parameters. Many precautions must be considered when dealing with hyperthermia such as the fluid containing magnetic nanoparticles are commonly water or saline and nanoparticles must be non-toxic and can work at the physiological pH. Also, MNPs must be tiny enough to be entrapped by cancer cells^28^. Another important parameter that determines the value of the induced heat and reproducibility of the obtained results is MNPs dispersion in solutions. Dispersion is essential to prevent MNPs precipitation and aggregation during the time of carrying out the experiment. In this context, highly dispersible graphene oxide sheets, GO, are used as platform to host and assemble MNPs on top of it. GO has high surface area and is biocompatible and by the functionalization of the MNPs through van der Waal force it is possible to attach MNPs to the GO sheets^29^. Ferrites are a class of ferrimagnetic materials primarily composed of iron oxide, having the general formula MFe_2_O_4_, where M is divalent metal ions like Mn^2+^, Fe^2+^, Co^+^, Ni^2+^, Cu^2+^, Zn^2+^, Mg^2+^ or Cd^2+^. Their spinel structure allows for the distribution of cations among tetrahedral and octahedral sublattices, enabling precise tuning of magnetic and hyperthermia properties. The large number of possible combinations make ferrite highly versatile, offering broad relevance in applications that improve quality of life^30^. Engineering their properties involves adding two or more dopants such as divalent metal ions Mn and Zn, rare-earth ions, or other metal oxides to tailor structural, magnetic, and electrical properties. This include increasing coercivity, enhancing magnetization, or reducing power loss for high-frequency applications^31^. Careful optimization of divalent cation substitution, such as Mn within iron ferrite, has shown a remarkable increase of around 181% in specific absorption rate (SAR), reaching 510 kW/kg^32^. This enhancement upon Mn doping is attributed to the elevated saturation magnetization resulting from the appropriate cation distribution within the MNPs. Furthermore, the increase in spin relaxation time with higher Mn concentration also contributes to the increased SAR values as MNPs retain their moments for extended period before relaxing, leading to higher SAR values. Another important application of ferrites, that is widely investigated is photocatalytic wastewater remediation, in which doping can modulate the bandgap of ferrites, thus improving activity under visible light^33–36^. On the other hand, cobalt ferrite (CoFe_2_O_4_) is a particularly compelling material to explore; it is a spinel ferrite known for its outstanding structural and magnetic properties, including high saturation magnetization and robust magneto crystalline anisotropy^37^. Furthermore, its relatively high Curie temperature and robust structural stability up to 600 °C ensure thermal stability under physiological conditions, a critical requirement for the sustained therapeutic use of magnetic nanoparticles^38–40^. However, the biocompatibility and cytotoxicity of CoFe_2_O_4_ and other ferrite nanoparticles are critical concerns for biomedical applications, such as magnetic hyperthermia^41^. Therefore, functionalizing ferrites with materials like chitosan, PEG, graphene, or graphene oxide (GO)^42^ is a vital strategy to ensure biological safety, while also enhancing dispersibility in solutions^43^.

In this work, MnFe_2_O_4_ and CoFe_2_O_4_ MNPs and their composites with GO were synthesized in one pot hydrothermal reaction. CoFe_2_O_4_ and MnFe_2_O_4_ are both ferrimagnetic compounds but have different magnetic behaviors, MnFe_2_O_4_ being classified as “soft ferrite” and CoFe_2_O_4_ as “hard ferrite” and this is attributed to the different inversion degree characterizing each one of these ferrites. While bulk cobalt ferrite is considered to display the completely inverse spinel structure, with all Co (II) ions exclusively at octahedral lattice sites, bulk MnFe_2_O_4_ adopts an almost normal spinel structure with a low degree of inversion^44,45^ corresponding to the formula (Mn_0.8_Fe_0.2_) [Mn_0.2_Fe_1.8_]O_4_. The variation in the magnetic structures and properties will boost variation on other parameters that could benefit applications like hyperthermia. This includes, but not only limited to, magnetic anisotropy, *k*, magnetic parameters like saturation magnetization, *M*_*s*_, area of hysteresis loop, *A*, remnant magnetization, M_r_, and superparamagnetic behavior. The choice of manganese ferrite NPs is due to its low toxicity, high stability, easily synthesized and tunable magnetic properties^46^. Many techniques are currently developed and used for the synthesis of MNPs^47–49^ which in turn allows engineering of many parameters that are required for the intended applications. Most of the investigations on magnetic nanoparticles have been carried out on spherical shaped nanoparticles, although other shapes (rods, disks, cubes, rhombohedral, or more complex shapes) have also been successfully synthesized. And in many cases mixed morphologies results as a final product that is used and assessed. We show experimentally that the difference in effective surface anisotropy between different NPs geometries leads to substantial differences in magnetic and anisotropy properties. However, the efficiency of hyperthermia as a therapy depends also, and to a large extent, on the MNPs’ physical characteristics: types, sizes and shapes^50^. Some reported values of SAR for cuboid/cubes of 17.6 nm-sized Mn based ferrite was found to be 57.2–278.7 W/g under fields of 250–750 Oe, such values depend primarily on the applied field strength, frequency and particle size^51^. The combination of shape / morphology and magnetocrystalline anisotropies can lead to a complex dependence of these parameters on magnetic hyperthermia which may also depend on the specific material and crystalline structure^43,48,52–56^.

## Experimental

### Synthesis of pure MnFe_2_O_4_ and GO-MnFe_2_O_4_ nanoparticles

MnFe_2_O_4_ nanoparticles were synthesized using 0.05 M Iron (III) Nitrate Nonahydrate (Aldrich, ≥ 98%) and 0.025 M Manganese (II) chloride tetra hydrate (Aldrich, 98%). Precursors were dissolved in 100 ml d. water and stirred for 30 min till the solution became clear. Then the pH of the previous mixture was adjusted to reach pH equal to 10 by adding NaOH (WINLAB, 98%) solution drop wise while stirring. After that, to get homogeneous solution, the mixture was stirred for another 15 min. The mixture was heated to 160 °C for 20 h with heating rate of 4 °C /min inside a Teflon lined stainless steel autoclave. The Teflon lined stainless was naturally cooled down to room temperature. Finally, the obtained powder was washed by using Centrifugation at 8000 rpm for 10 min with deionized waters, this washing procedure was repeated 3 times to get rid of the undispersed residue. The final washing was done using ethanol. Finally, the as obtained MNPs were dried at 80 °C in the oven. For the preparation of Go-MnFe_2_O_4_, GO (Aldrich) was added to 40 ml d. water and sonicated for at least for 40 min to get a dispersed solution. Other precursors, namely, Iron (III) Nitrate Nonahydrate and Manganese (II) chloride tetra hydrate were added to 60 ml d. water, with stirring for 30 min. Both solutions were mixed and stirred for another 30 min. Final thermal treatment was done as for the MnFe_2_O_4_. Case.

### Synthesis of pure CoFe_2_O_4_and GO-CoFe_2_O_4_ nanoparticles

CoFe_2_O_4_ nanoparticles were synthesized using 1.4 × 10^− 2^ M cobalt (II) chloride (Fluke, ≥ 98%) and 0. 2.8 × 10^− 2^ M Iron acetyl acetonate (Aldrich, 97%). Precursors were dissolved in 100 ml di-water and stirred for 1 h. till the solution became clear. The pH of the previous mixture was adjusted to 11 by adding NaOH solution drop wise while stirring till homogenous solution is obtained. The mixture was heated to 220 °C for 15 h with heating rate 4 °C/min inside a Teflon lined stainless steel autoclave then cooled down to room temperature naturally. Finally, the obtained powder was washed, using d. water, several times by Centrifugation. The final washing was done using ethanol and the as obtained MNPs were dried at 80 °C in the oven. For the preparation of GO-CoFe_2_O_4_, GO (Aldrich) was added to 70 ml d. water and sonicated at least for 1 h to get a dispersed solution. Cobalt (II) chloride and Iron acetyl acetonate added to 30 ml di-water and stirred for 30 min. Both solutions were mixed and stirred for another 30 min hence other steps are similar as the above method.

### Characterization of the nanoparticles

X-Ray powder diffraction analysis was carried out using a Panalytical X-ray diffractometer with Cu Kα target having radiation (λ = 1.5405 Å) for all samples. The crystallite size of nanoparticles was estimated from the peaks broadening using the well-known Scherer formula. Transmission electron microscope (TEM) images were taken using a JSM1400-PLUS-JEOL. Samples were prepared by placing a drop of the particles suspended in ethanol onto a carbon coated copper grid and allowing it to dry at room temperature. Particles distribution was obtained from TEM by manual measurement of the largest dimension along two perpendicular directions of more than 300 particles using ImageJ software. Vibrating sample magnetometer (VSM) (Quantum Design, 3T Versalab) was used to study magnetic properties. Feasibility for hyperthermia of 5 mg of the nanoparticles dispersed in 1 mL of water was measured using G2-D5 Series Multi-mode 1500 W Driver from Nanoscale Bio magnetics. The sample solution was placed into the G2-D5 coil, where the alternating magnetic field amplitude and frequency can be applied. The induced temperature of the solution was detected and recorded using a fiber-optic sensor. Raman spectra were measured at 532 nm by using a Confocal Raman microscope (WItec, 300R alpha, made in Germany) with Laser power 1mw. Fourier Transform Infrared (FT-IR) spectroscopy was conducted using a PerkinElmer Spectrum 2B (USA) spectrophotometer within the wavenumber range of 400–4000 cm^−1^ to analyse the structural features of PPACs nanocomposites, prepared in the form of potassium bromide (KBr) pellets. The Raman spectra and images presented in this work were recorded using a Confocal Raman Microscope (alpha 300 R from WITec GmbH, Ulm, Germany) equipped with a Nd-YAG laser working at 532 nm and an ultrahigh throughput spectrometer (UHTS 300) for Raman light analysis. FTIR spectra were recorded using (FT/IR-4600, JASCO Corp., Tokyo, Japan) FTIR spectrometer.

## Results and discussion

### Crystal structure and morphology

**Fig. 1:**
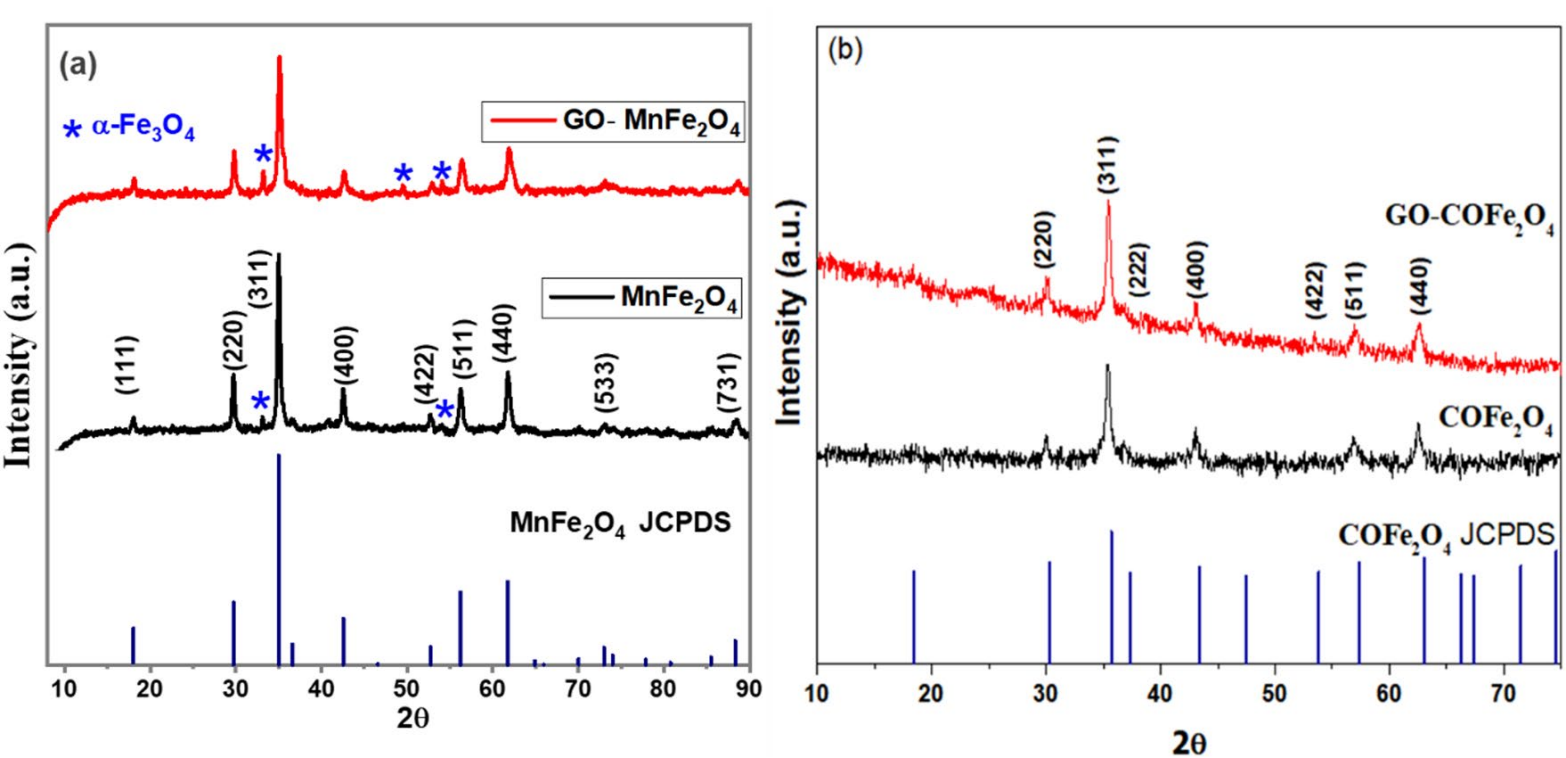
X-ray diffraction pattern of (**a**) MnFe_2_O_4_, GO-MnFe_2_O_4_ nanocomposite and the standard for MnFe_2_O_4_ (**b**) CoFe_2_O_4_, GO-CoFe_2_O_4_ nanocomposites and the standard for CoFe_2_O_4_.

The XRD patterns of MnFe_2_O_4_, GO-MnFe_2_O_4_ and the standard MnFe_2_O_4_ pattern is shown in Fig. 1a. The peak positions and relative intensities match well with the standard XRD data for MnFe_2_O_4_ cubic spinel structure. The XRD pattern of MnFe_2_O_4_ exhibits diffraction peaks at 2θ = 18.09°, 29.78°, 35.08°, 36.68°, 42.6°, 52.77°, 56.3°, 61.8°, 73.06°, and 88.4°, corresponding to the (111), (220), (311), (222), (400), (422), (511), (440), (533), and (731) crystal planes of the spinel ferrite phase, respectively^57,58^. Attention was directed to the (311) reflection, as this peak is widely used as a diagnostic marker for lattice parameter variation, cation redistribution, and defect formation in spinel ferrites. The position of the (311) peak is highly sensitive to the occupancy of tetrahedral (A) and octahedral (B) sites by Mn^2+^ and Fe^3+^ ions, since ionic radii differences between these cations directly modulate the d-spacing of this reflection. The observed position at 2θ = 35.08° in bare MnFe_2_O_4_ is consistent with a partially inverse spinel configuration characteristic of hydrothermally synthesized MnFe_2_O_4_^57,58^. Upon incorporation into the GO composite, the (311) reflection shifts to 2θ = 35.73°, corresponding to a shift of + 0.65°. Using Bragg’s law, this shift corresponds to a reduction in the (311) interplanar spacing from approximately 2.556 Å in bare MnFe_2_O_4_ to approximately 2.512 Å in the MnFe_2_O_4_-GO composite, representing a lattice contraction of roughly 1.7%. This contraction is indicative of compressive macrostrain introduced at the ferrite–graphene oxide interface, likely arising from electrostatic and coordinative interactions between surface metal cations and the oxygen-bearing functional groups of GO (hydroxyl, epoxide, and carboxyl groups). Such interfacial interactions can locally perturb the cation distribution near the nanoparticle surface, promoting partial migration of cations between A and B sites and thereby inducing measurable lattice distortion. Notably, minor peaks at 2θ = 33.07°, 49.5°, and 54.20° present in bare MnFe_2_O_4_ were indexed to the hematite phase (α-Fe_2_O_3_)^59,60^, suggesting incomplete phase purity under the hydrothermal conditions used. Notably, when MnFe_2_O_4_ is combined with graphene oxide (GO) to form GO-MnFe_2_O_4_, the intensity of hematite peaks increased further which could be related to the presence of GO functional group. Those functional groups can interact with the iron precursors, altering the reaction pathway and leading to the presence of different iron oxide phases^61^. All XRD peaks in Fig. 1a are broad, indicating small crystalline size. The average crystallite size of both samples was calculated from the five most intense reflections using the Scherrer equation (Eq. 1) and was found to be 22.7 ± 2.8 nm and 18.5 ± 2.1 nm for MnFe_2_O_4_ and GO-MnFe_2_O_4_, respectively.

1 $$\:\boldsymbol{D}=\frac{0.93\:\boldsymbol{\lambda\:}}{\boldsymbol{\beta\:}\mathbf{cos}\boldsymbol{\theta\:}}$$

where D is the average crystallite size, λ is the X-ray wavelength (Cu-Kα, 1.5406 Å), β is the full width at half-maximum FWHM of the diffraction peak, and θ is the Bragg diffraction angle. While the Scherrer equation provides a convenient first-order estimate of crystallite size, it assumes that peak broadening arises solely from crystallite size effects and does not account for additional broadening contributions that are commonly present in nanostructured ferrites. Microstrain arising from lattice distortions, non-uniform cation distributions, and structural defects can contribute significantly to peak broadening and lead to an underestimation of true crystallite size if not corrected for. In the present system, the observed reduction in crystallite size from 22.7 ± 2.8 nm in bare MnFe_2_O_4_ to 18.5 ± 2.1 nm in GO-MnFe_2_O_4_ is consistent with the lattice contraction evidenced by the (311) peak shift discussed above, and likely reflects a combination of true crystallite size reduction and enhanced micro strain at the ferrite–GO interface. The oxygen-bearing functional groups of GO can interact with surface metal cations, introducing local lattice distortions that broaden diffraction peaks beyond what would be expected from size effects alone. Given the moderate number of well-resolved peaks available, the Scherrer values are reported as primary estimates with the aforementioned caveats acknowledged.

XRD patterns of the synthesized CoFe_2_O_4_ and GO-CoFe_2_O_4_ nanoparticles are shown in Fig. 1b. The observed diffraction peaks located at 2θ = 30.02°, 35.73°, 36.86°, 42.8°, 53.29°, 56.9°, and 62.54° are assigned to the (220), (311), (222), (400), (422), (511), and (440) planes of the CoFe_2_O_4_ phase, matching well with the standard single-phase spinel structure of CoFe_2_O_4_ (JCPDS no. 22-1086). The XRD patterns show that the characteristic peak of graphene oxide is not observable, indicating that GO incorporation does not significantly perturb the crystalline structure of CoFe_2_O_4_, consistent with the dominant diffraction signals of the spinel phase masking any residual GO reflection. The average crystallite size calculated from the FWHM using Eq. (1) was found to be 13.9 ± 1.5 nm and 14.7 ± 1.8 nm for CoFe_2_O_4_ and GO-CoFe_2_O_4_, respectively. Unlike the MnFe_2_O_4_ system, where GO incorporation produced a measurable reduction in crystallite size, the CoFe_2_O_4_ composite exhibits a marginal size increase of approximately 0.8 nm. This behavior may reflect the higher magnetic anisotropy and greater lattice rigidity of CoFe_2_O_4_, which render its crystalline structure comparatively less susceptible to interfacial strain-induced size suppression. Nevertheless, the same caveats regarding Scherrer-based size estimation apply here: micro strain contributions from Co^2+^/Fe^3+^ cation disorder across tetrahedral and octahedral sites, as well as instrumental broadening, may partially convolute the apparent crystallite size values reported for both CoFe_2_O_4_ and its GO composite. The close similarity between the two CoFe_2_O_4_ values further suggests that GO has a minimal effect on the crystallization behavior of the hard ferrite phase under the hydrothermal conditions employed.

**Fig. 2:**
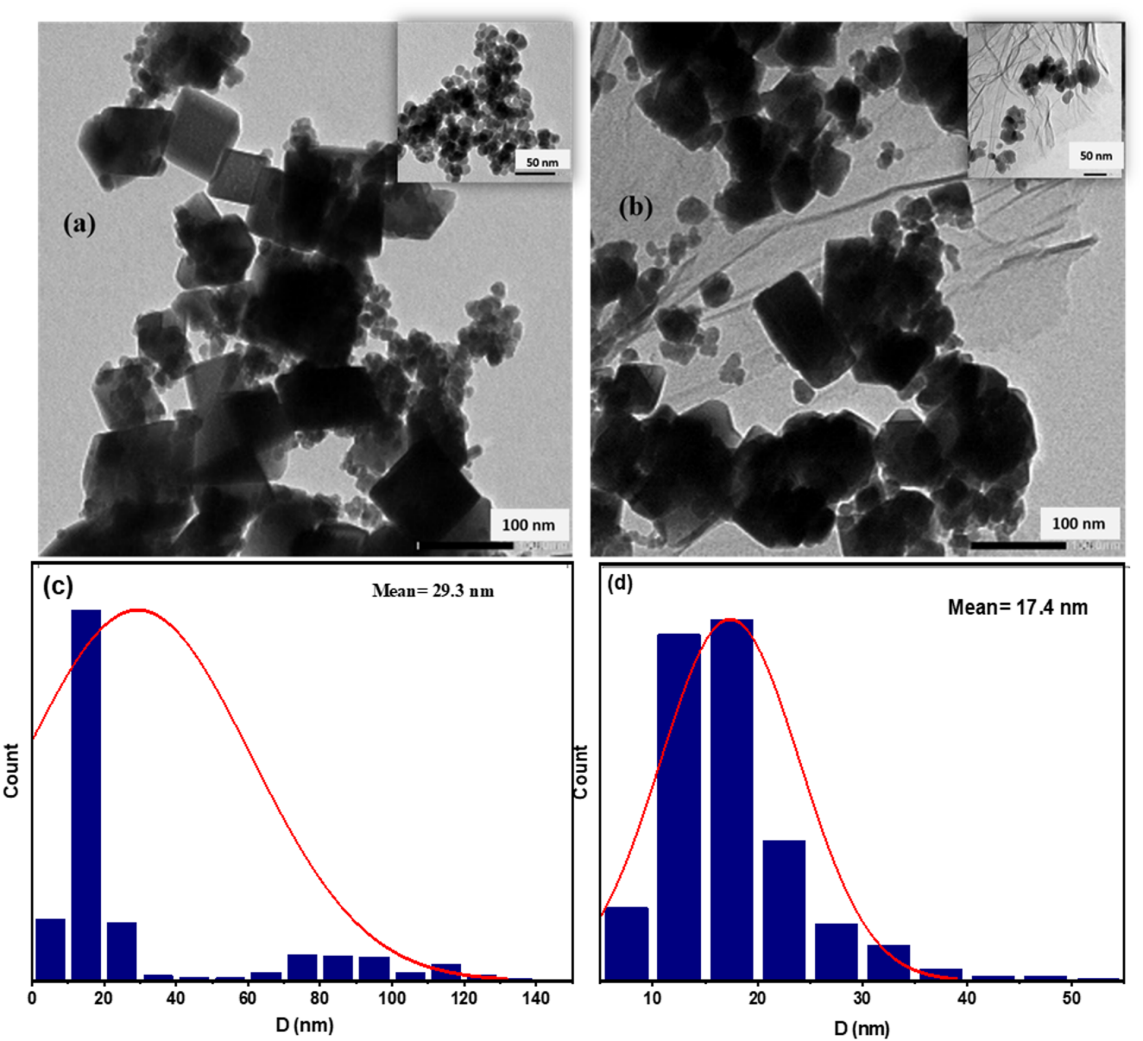
TEM image for (**a**) MnFe_2_O_4_ (**b**) GO-MnFe_2_O_4_. The insets to fig. (**a**,**b**) show TEM image at lower magnification. Fig. (**c**,**d**) represents the size distribution for MnFe_2_O_4_ and GO-MnFe_2_O_4_ nanoparticles, respectively.

Transmission electron microscope (TEM) images of both MnFe_2_O_4_ and GO-MnFe_2_O_4_ nanocrystals are shown in Fig. 2a,b, respectively. Figure 2a shows the well-defined MnFe_2_O_4_ particles which exhibit mixed morphology; cubic and/or cuboid with an edge length of around 14.9 nm and spheres (but to a lesser extent) (inset to Fig. 2a. The TEM images of GO-MnFe_2_O_4_ (Fig. 2b) show the formation of more spherical particles when compared to MnFe_2_O_4_. It can be clearly seen that two-dimensional GO nanosheets are exfoliated and MnFe_2_O_4_ nanoparticles are assembled on top of the sheets. Figure 2c,d show the corresponding histogram plots of the sizes using ImageJ analysis of both MnFe_2_O_4_ and GO-MnFe_2_O_4_, respectively. From the histogram, the calculated average particle size is 29.3 with standard deviation (SD) of ± 8 nm and 17.4 with SD of ± 4 nm for MnFe_2_O_4_ and GO-MnFe_2_O_4_, respectively. And as expected, particle size calculated from TEM is larger than those obtained from the XRD measurement. Morphology and particle size could significantly influence the material hyperthermia performance and SAR values^54^.

**Fig. 3:**
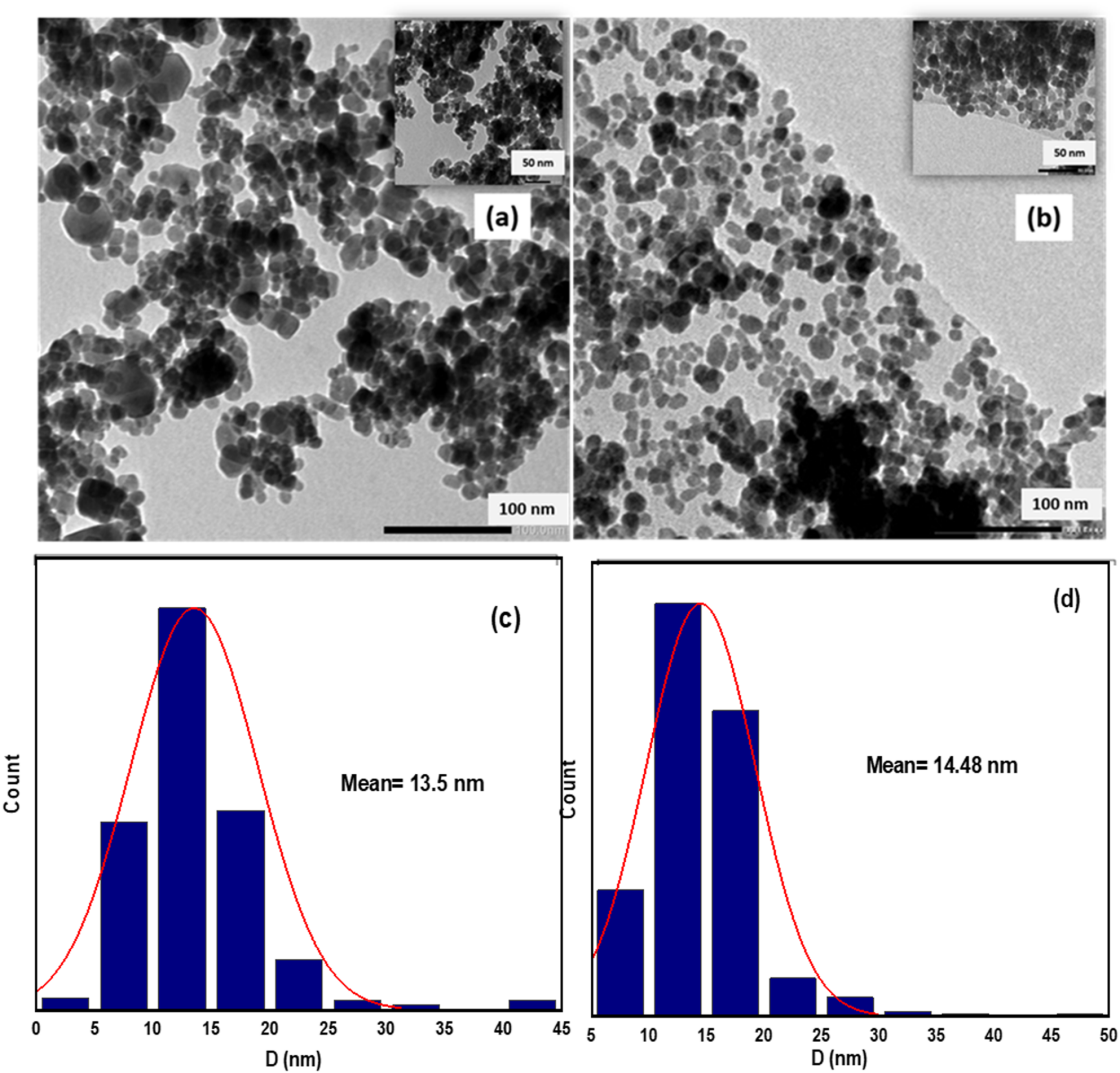
TEM image for (**a**) CoFe_2_O_4_ (**b**) GO-CoFe_2_O_4_. The insets to fig. (**a**,**b**) show TEM image at lower magnification. Fig. (**c**,**d**) represents the size distribution for CoFe_2_O_4_ and GO-CoFe_2_O_4_ nanoparticles, respectively.

Figure 3a,b show TEM for CoFe_2_O_4_ and GO-CoFe_2_O_4_, respectively. Both samples show spherical particles having average particle size of 13.5 with SD of ± 3.4 nm and 14.48 nm with SD of ± 3.8 nm, respectively. In the case of the GO-CoFe_2_O_4_ composite, particles were disbursed on the surface of the 2D GO sheets signifying a successful attachment of the ferrite nanoparticles on GO. Figure 3c,d show the corresponding histogram plots of CoFe_2_O_4_ and GO-CoFe_2_O_4,_ respectively. One must mention here that the synthesis temperature and time used for CoFe_2_O_4_ were 220 °C and 15 h. respectively, which is different from those used in case of MnFe_2_O_4_, 160 °C and 20 h. The use of high temperature could truncate and smooth the particles edges, so a spherical shape is more likely to form in comparison to the synthesis at lower temperature^62^. On the other hand, the longer reaction time allows for the Ostwald ripening of the small particles, which are then dissolved and incorporated into the larger ones contributing to a broader size distribution^62–65^. In the case of CoFe_2_O_4_, when we used similar temperature and time to that of MnFe_2_O_4_, we observed a wide hysteresis loop with large value of coercive filed, H_c_, which is not recommended for hyperthermia application.

**Fig. 4:**
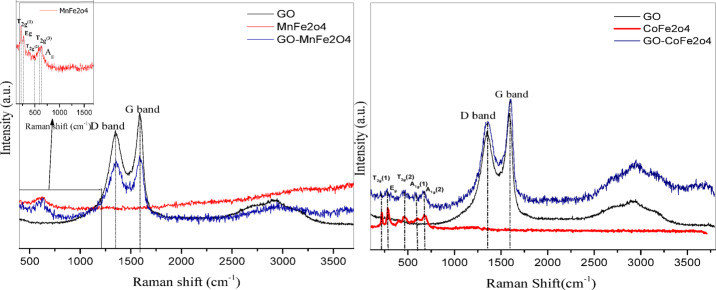
Raman (**a**) for GO, GO-MnFe_2_O_4_ and MnFe_2_O_4_ and (**b**) for GO, GO-CoFe_2_O_4_ and CoFe_2_O_4_.

Raman spectroscopy is a commonly used tool for the characterization of carbon products since conjugated and double carbon-carbon bonds lead to high Raman intensities. Figure 4a shows Raman spectra of GO, MnFe_2_O_4_ and GO-MnFe_2_O_4_. It shows the two fundamental bands of GO: D at 1347 cm^− 1^ arising from the sp^3^ carbon atoms of disordered graphite and G at 1588 cm^− 1^ arising from sp^2^- ordered graphite^66^. The I_D_/I_G_ ratio was found to be 0.99. On the other hand, the active Raman bands observed at 636 cm^− 1^, 270, 210, 484 and 585 cm^− 1^ are assigned to A_g1_, E_g_ and 3T_2g_ of the MnFe_2_O_4_ structure respectively.^67^. Figure 4b shows Raman spectra of GO, CoFe_2_O_4_ and GO-CoFe_2_O_4_. As stated earlier in the case of Mn Ferrite and GO-Mn, two fundamental bands of GO: D at 1355 cm^− 1^ and G at 1603 cm^− 1^ and the I_D_/I_G_ ratio was found to be 0.94. This calculated I_D_/I_G_ value is slightly less than that of the GO-MnFe_2_O_4_ case, indicating presence of less defects. Bands at 668, 589, 454, 273 and 217 are assigned to A_1g_ (2), A_1g_ (1), T_2g_ (2) E_g_ and T_2g_ (1) for the CoFe_2_O_4_, respectively^68,69^. Raman results verify the successful synthesis of the MnFe_2_O_4_, CoFe_2_O_4_ and the structural constancy of graphene oxide in the composites. In Raman spectroscopy the I_D_/I_G_ ratio measures the structural disorder and the density of defects/oxygen functional groups in the graphene lattice. In this context, the lower I_D_/I_G_ ratio for GO- CoFe_2_O_4_ composite compared to that of GO- MnFe_2_O_4_ indicates that the former has fewer defects, lower functional group density as well as a higher degree of graphitization. This is consistent with the XRD results, which show no reflections from impurity phases or defects in the cobalt composite, unlike the GO-MnFe_2_O_4_ composite, which contains the impurity phase hematite.

**Fig. 5:**
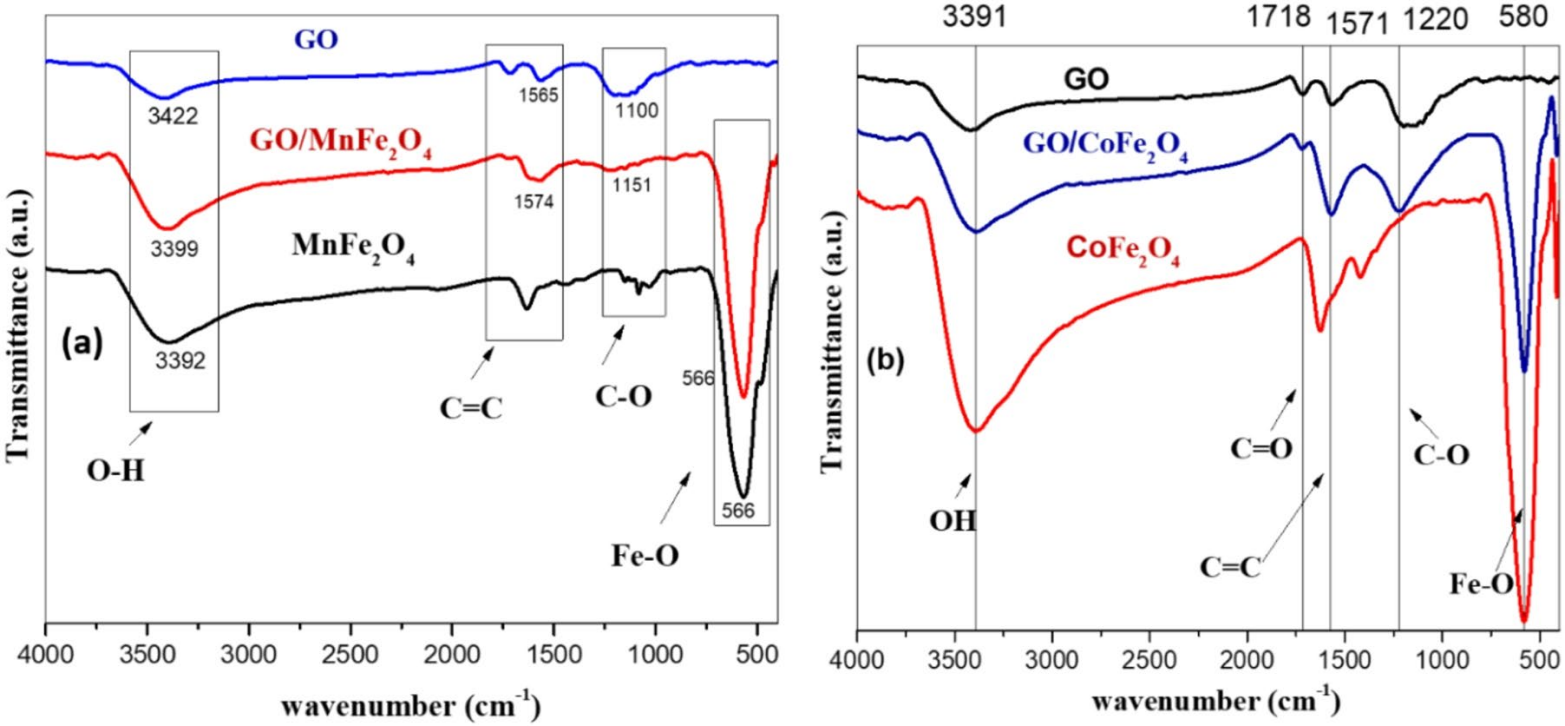
FTIR spectra of (**a**) GO, GO-MnFe_2_O_4_ and MnFe_2_O_4_ (**b**) for GO, GO-CoFe_2_O_4_ and CoFe_2_O_4_.

FTIR spectroscopy was used to investigate the effect of graphene oxide on ferrites NPS and to verify the integrity of the NPS. Figure 5a shows FTIR for MnFe_2_O_4_, GO-MnFe_2_O_4_ and GO for the purpose of comparison. It clearly indicates the formation of GO- MnFe_2_O_4_ composite. It’s observed a broad peak from (3392 cm^−1^-3422 cm^−1^ which is a fingerprint of Hydroxyl group stretching vibrations of adsorbed water. FTIR of GO-MnFe_2_O_4_ shows the vibrations of functional groups C=C Aromatic group and C–O epoxy group at 1574 and 1151, respectively^70^. In addition to the absorption peak at 566 cm^−1^ which represent the Fe–O stretching vibration in tetrahedral sites that only present in the spectra of MnFe_2_O_4_ and GO-MnFe_2_O_4_^71,72^. Figure 5b shows FTIR for CoFe_2_O_4_, obtained in the presence of graphene oxide. According to FTIR spectra exhibited a broad peak at approximately 3391 cm^−1^ which corresponds to the -OH Hydroxyl group, 1718 cm^−1^ C=O as a fingerprint for carboxyl group,1571 cm^−1^ which corresponds to C=C as Aromatic group and 1220 cm^−1^ which corresponds to C–O epoxy group^73^. In addition to the cobalt ferrite peak is observed at 580 cm^−1^ which represent the Fe–O stretching vibration in tetrahedral sites^71,72^. From the above mentioned FTIR spectroscopy, one can clearly identify abundant oxygen-containing functional groups on the surface of GO sheets like carboxyl, hydroxyl and epoxy groups, which make the surface of GO negatively charged. When GO suspension was poured into the metal cations (Mn and Co) solution, they are adsorbed on the surface of GO nanosheets through electrostatic attractions.

### Magnetic properties and hyperthermia measurements

CoFe_2_O_4_ and MnFe_2_O_4_ are both ferrimagnetic compounds but exhibit distinctly different magnetic behavior: MnFe_2_O_4_ is classified as a “soft ferrite” while CoFe_2_O_4_ is classified as a “hard ferrite,” a distinction that arises primarily from the different inversion degrees characterizing each spinel structure^74^. The magnetocrystalline anisotropy constant (K_1_) of CoFe_2_O_4_ is reported in the range of 2.0–3.0 × 10^5^ J/m^3^, whereas MnFe_2_O_4_ exhibits a much lower K₁ of approximately − 0.36 × 10^5^ J/m^3^ ^75^, reflecting the dominant role of Co^2+^ ions occupying octahedral sites in generating strong uniaxial anisotropy. One cannot ignore the surface and finite size effects that are known to play a key role in the magnetic properties of nanoparticles^76,77^. A direct correlation between magnetization and particle size has been reported in several ferrite systems^48,53,54^, making it important to establish this effect for the present composites.

**Fig. 6:**
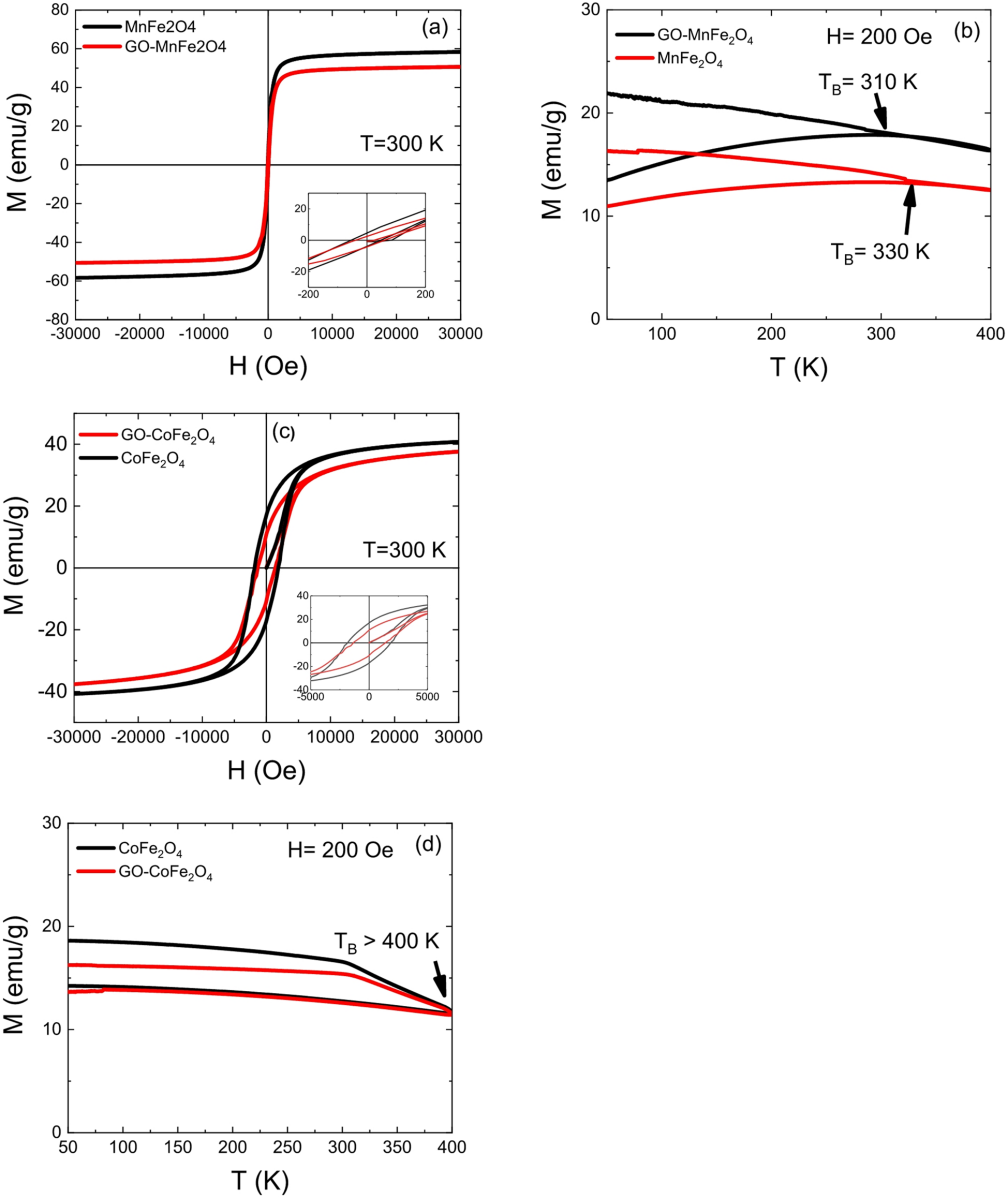
(**a**,**c**) magnetic hysteresis loops of ferrite system at 300 k for GO-MnFe_2_O_4_, MnFe_2_O_4_ and GO-CoFe_2_O_4_, CoFe_2_O_4_, respectively. (**b**,**d**) ZFC-FC magnetization curves taken under 200 Oe for GO-MnFe_2_O_4_, MnFe_2_O_4_ and GO-CoFe_2_O_4_, CoFe_2_O_4_, respectively.

The room-temperature M×H loops of the standalone Co and Mn ferrites and their GO composites were measured and are shown in Fig. 6a–c. For MnFe_2_O_4_ and GO-MnFe_2_O_4_ nanoparticles, nearly zero remanence magnetization is observed at 300 K, confirming superparamagnetic behavior at room temperature. The measured saturation magnetization (M_S_) values of 60 emu/g and 50 emu/g, and coercive fields of HC ≈ 65 Oe and ≈ 50 Oe for MnFe_2_O_4_ and GO-MnFe_2_O_4_, respectively (Fig. 6a, are consistent with values reported in the literature for hydrothermally synthesized MnFe_2_O_4_ nanoparticles of comparable size. Specifically, M_S_ values in the range of 38–64 emu/g and H_C_ values below 100 Oe have been widely reported for hydrothermally synthesized MnFe_2_O_4_ nanoparticles in the 15–30 nm size range^78,79^, confirming that our samples fall within the expected magnetic parameter space for this system. The M_S_ value of 60 emu/g obtained here is notably lower than the bulk value of 82 emu/g^80^, a reduction of approximately 27%, This reduction is consistent with the well-established surface spin canting effect in which the noncollinear arrangement of magnetic spins at the nanoparticle surface creates a magnetically disordered shell that does not contribute to the net magnetization^81^. The further reduction to 50 emu/g upon GO incorporation (a decrease of ~ 17% relative to bare MnFe_2_O_4_) is in line with reports on similar ferrite–GO and ferrite–carbon matrix composites, where the nonmagnetic matrix dilutes the magnetic phase and introduces additional surface disorder through interfacial metal–oxygen interactions^82,83^.

The hysteresis loops of CoFe_2_O_4_ and GO-CoFe_2_O_4_ nanoparticles (Fig. 6c) exhibit the characteristic features of hard ferromagnetic materials. The measured coercivity values of H_C_ ≈ 2.5 kOe and ≈ 2.0 kOe, and MS values of ≈ 40 emu/g and ≈ 35 emu/g for CoFe_2_O_4_ and GO-CoFe_2_O_4_, respectively. These values are in good agreement with literature reports for CoFe_2_O_4_ nanoparticles in the 10–20 nm size range synthesized by hydrothermal methods, where H_C_ values of 1.5–3.0 kOe and M_S_ values of 35–55 emu/g are commonly reported^84,85^. The measured M_S_ of 40 emu/g represents a reduction of 50% relative to the bulk value of 80 emu/g^86^, a larger relative reduction than observed for MnFe_2_O_4_ (27%). This more pronounced suppression of magnetization in CoFe_2_O_4_ nanoparticles is consistent with their higher surface anisotropy and greater sensitivity to surface spin disorder, which is amplified in high-anisotropy systems where canted surface spins are more strongly pinned and thus more effectively excluded from contributing to the net moment^87^. The coercivity of 2.5 kOe observed for our CoFe_2_O_4_ sample is within the broad range reported for this system (0.5–5.0 kOe), with the specific value reflecting a combination of particle size, synthesis conditions, and degree of cation inversion^84^. The reduction in H_C_ from 2.5 kOe to 2.0 kOe upon GO incorporation is attributed to surface anisotropy dilution caused by the non-magnetic GO matrix interfering with the coherent spin reversal mechanism, an effect also reported by Venkatesha et al. for CoFe_2_O_4_–graphene oxide composites^82^. As expected, the M_S_ values of both ferrite systems are significantly lower than their respective bulk counterparts, as bulk CoFe_2_O_4_ and MnFe_2_O_4_ exhibit M_S_ values of 80 emu/g^86^ and 82 emu/g^80^, respectively. The reduction in magnetization with decreasing particle size is a well-documented consequence of increased surface-to-volume ratio: as particle size decreases, the proportion of surface spins exhibiting canted or disordered configurations increases, leading to a net reduction in magnetization^81^. Since GO is nonmagnetic, it contributes negatively or not at all to the overall magnetization, further lowering M_S_ in the composite samples.

Figure 6b,d show the zero-field-cooled (ZFC) and field-cooled (FC) magnetization as a function of temperature at a constant applied field of 200 Oe. The ZFC/FC bifurcation and the peak in the ZFC curve define the blocking temperature (T_B_), which marks the transition between the superparamagnetic (fast relaxation) and blocked (slow relaxation) regimes. T_B_ values of 310 K and 330 K were obtained for MnFe_2_O_4_ and GO-MnFe_2_O_4_, respectively. The slight increase in T_B_ upon GO incorporation is consistent with enhanced interparticle dipolar interactions mediated by the GO matrix. Such interaction can restrict nanoparticle moment relaxation and effectively raise the energy barrier for spin reversal, an effect well established in nanoparticle–matrix systems where reduced inter-particle spacing or physical confinement strengthens dipolar coupling. The T_B_ values near room temperature for the MnFe_2_O_4_ system are in good agreement with reported values for MnFe_2_O_4_ nanoparticles of similar size^78,79^, and are consistent with the superparamagnetic behavior evidenced by the near-zero coercivity observed in the room-temperature hysteresis loops. For CoFe_2_O_4_ and GO-CoFe_2_O_4_, T_B_ > 400 K, well above room temperature, consistent with the high magnetocrystalline anisotropy of CoFe_2_O_4_ that stabilizes magnetic domains against thermal fluctuations up to elevated temperatures.

**Fig. 7:**
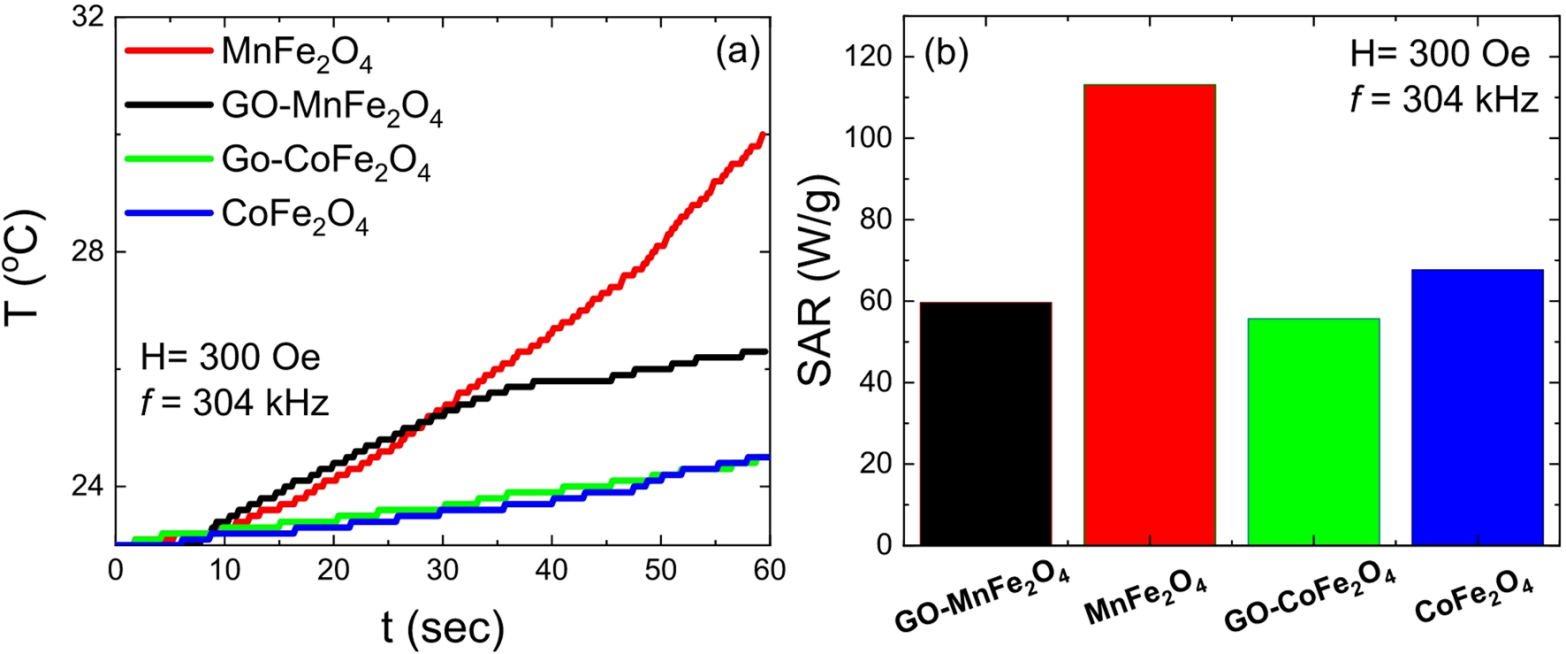
(**a**) Hyperthermia temperature curves for GO-MnFe_2_O_4_, MnFe_2_O_4_, GO-CoFe_2_O_4_ and CoFe_2_O_4_ in concentration of 5 mg/mL in water under field application of 300 Oe and 304 kHz (**b**) Calculated specific absorption rate (SAR) from initial slope of T × t curve for GO-MnFe_2_O_4_, MnFe_2_O_4_, GO-CoFe_2_O_4_ and CoFe_2_O_4_ nanoparticles.

This is in agreement with the literature, where T_B_ values exceeding 400 K are routinely reported for CoFe_2_O_4_ nanoparticles even at sizes below 20 nm^79,80^, reflecting the dominant role of anisotropy energy over thermal energy in this system. Figure 7a shows the temperature increase as a function of time for both ferrites and their GO composites suspended in water at a concentration of 5 mg/mL under an alternating magnetic field of 300 Oe (23.9 kA/m) and frequency of 304 kHz. All samples can generate heat under these conditions. MnFe_2_O_4_ shows a significantly greater temperature increase compared to the GO-MnFe_2_O_4_ composite, which is attributed to the reduced magnetization of the composite upon GO incorporation. In the case of CoFe_2_O_4_ and GO-CoFe_2_O_4_, a smaller temperature increase is observed in both cases with approximately the same shape of the heating curves. These results can be understood in terms of the distinct magnetic properties of the two ferrite systems, and particularly the role of magnetic anisotropy in determining which relaxation mechanism dominates heat dissipation under the experimental field conditions applied here. The magnetocrystalline anisotropy constant K_eff_ is 3.3 × 10^3^ J/m^3^ for MnFe_2_O_4_ and 2.7 × 10^5^ J/m^3^ for CoFe_2_O_4_^88^, a difference of nearly two orders of magnitude, and this contrast is central to understanding the divergent hyperthermia behavior of the two systems. The induced heating mechanism arises from energy dissipation by magnetic nanoparticles under an alternating magnetic field (AMF), and two major relaxation pathways contribute to this dissipation. The first is Néel relaxation, in which the internal magnetization rotates between easy axes within a magnetically blocked particle without physical rotation of the particle itself. The Néel relaxation time is given by

$$\:{\tau\:}_{N}={\tau\:}_{0}\mathrm{e}\mathrm{x}\mathrm{p}\left(\frac{KV}{{k}_{B}T}\right)$$

where τ_o_ ~ 10^−10^ s is the characteristic attempt time, K is the anisotropy constant, V is the particle volume, k_B_ is the Boltzmann constant, and T is the absolute temperature. The argument of the exponential, KV/k_B_T, is the ratio of the anisotropy energy barrier to thermal energy and is the decisive parameter governing whether Néel relaxation is efficient under a given AMF frequency. The second is Brownian relaxation, in which the entire nanoparticle physically rotates within the carrier fluid, dragging its magnetic moment with it. The Brownian relaxation time is $\:{\tau\:}_{B}=\frac{3\eta\:{V}_{h}}{{k}_{B}T}$, where η is the dynamic viscosity of the carrier fluid and V_h_ is the hydrodynamic volume of the nanoparticle. Brownian relaxation is independent of the anisotropy constant K and depends instead on particle size and the viscous properties of the medium. In a real suspension, the effective relaxation time τ_eff_ is given by the harmonic mean of the two contributions $\:\frac{1}{{\tau\:}_{eff}}=\frac{1}{{\tau\:}_{N}}+\frac{1}{{\tau\:}_{B}}$.

Maximum heating efficiency is achieved when τ_eff_ ≈ 1/(2πf), i.e., when the effective relaxation time is matched to the period of the applied AMF. At the experimental frequency of 304 kHz, the optimal relaxation time is approximately τ_opt_ ≈ 5.2 × 10^−7^ s. For MnFe_2_O_4_, with K = 3.3 × 10^3^ J/m^3^ and a crystallite size of approximately 24 nm (V ≈ 7.24 × 10^−24^ m^3^), the Néel energy barrier KV/k_B_T at room temperature (T = 300 K) is approximately

$$\:\frac{KV}{{k}_{B}T}=\frac{3.3\times\:{10}^{3}\times\:7.24\times\:{10}^{-24}}{1.38\times\:{10}^{-23}\times\:300}\approx\:5.77$$

giving a Néel relaxation time of τ_N_ ≈ τ₀_o_exp(5.77) ≈ 3.2 × 10^−8^ s. This value is short relative to the AMF period (τ_opt_ ≈ 5.2 × 10^−7^ s), indicating that the magnetization of MnFe_2_O_4_ can follow the AMF with relatively low lag, and that Néel relaxation is fast and efficient. Under the aqueous suspension conditions of this experiment (η_water_ ≈ 8.9 × 10^−4^ Pa s), the Brownian relaxation time for a particle with a hydrodynamic diameter of ~ 30 nm (accounting for a hydration shell) is τ_B_ ≈ 3 × 8.9 × 10^−4^ × (π/6)(30 × 10^−5^)^3^/(1.38 × 10^−23^ × 300) ≈ 3.8 × 10^−5^ s. Since τ_N_ and τ_B_ are of similar magnitude for MnFe_2_O_4_, both Néel and Brownian mechanisms operate concurrently, with τ_eff_ falling in the range 1–2 × 10^−5^ s. While this is still somewhat shorter than τ_opt_, the low-anisotropy character of MnFe_2_O_4_ means the energy barrier per cycle is modest, allowing the magnetization to respond rapidly and efficiently to the oscillating field, resulting in appreciable hysteretic losses per cycle and the higher SAR values observed. For CoFe_2_O_4_, with K = 2.7 × 10^5^ J/m^3^ and a crystallite size of approximately 13.9 nm (V ≈ 1.40 × 10^−24^ m^3^), the Néel energy barrier is

$$\:\frac{KV}{{k}_{B}T}=\frac{2.7\times\:{10}^{5}\times\:1.40\times\:{10}^{-24}}{1.38\times\:{10}^{-23}\times\:300}\approx\:91.3$$

giving τ_N_ ≈ 10^−10^ × exp(91.3) ≈ 10^−10^ × 10^39.7^ s, an astronomically large value that is many orders of magnitude beyond any physically accessible timescale. This result has a critical implication: Néel relaxation is completely frozen out in CoFe_2_O_4_ at room temperature under these field conditions. The anisotropy barrier is so large that internal magnetization reversal cannot occur within the AMF period, and the particle behaves as a magnetically blocked, single-domain particle. This is consistent with the conventional hard-magnetic hysteresis loop observed in Fig. 6c and the T_B_ > 400 K measured from ZFC/FC curves. Since Néel relaxation does not contribute to heating in CoFe_2_O_4__4_, Brownian relaxation is the only active heating mechanism in this system. However, because τ_eff_ ≈ τ_B_ ≈ 10^−8^ s for particles of this size in water, which is still significantly shorter than τ_opt_ ≈ 5.2 × 10^−7^ s, the Brownian mechanism also operates sub-optimally, leading to reduced energy absorption per cycle and consequently lower SAR values relative to MnFe_2_O_4_. The near-identical heating curves of CoFe_2_O_4_ and GO-CoFe_2_O_4_ further support this interpretation: since heating in this system arises entirely from viscous (Brownian) rotation rather than internal magnetization dynamics, the nonmagnetic GO matrix, which does not significantly alter the particle’s hydrodynamic volume or the suspension viscosity at this loading, has a comparatively minor effect on τ_B_ and hence on SAR, explaining why the two CoFe_2_O_4_-based samples show similar temperature rise profiles.

In this light, the superior SAR of MnFe_2_O_4_ (110 W/g) relative to CoFe_2_O_4_ (70 W/g) under the experimental conditions used here is a direct consequence of the anisotropy-mediated relaxation dynamics: MnFe_2_O_4_ benefits from concurrent Néel and Brownian contributions with relaxation times that are better matched to the applied frequency, while CoFe_2_O_4_ is limited to Brownian relaxation alone with its internal magnetization effectively locked along the easy axis. This analysis also clarifies why the two systems respond differently to GO incorporation. In the low-anisotropy MnFe_2_O_4_ system, where Néel relaxation contributes substantially, the introduction of GO reduces M_S_ and introduces domain-pinning defects (oxygen vacancies and residual α-Fe_2_O_3_ phase as evidenced from XRD), directly impairing the internal magnetization dynamics and causing a ~ 45% reduction in SAR (from 110 to 60 W/g). In contrast, in CoFe_2_O_4_ where only Brownian relaxation is operative, the GO matrix has minimal influence on τ_B_ at the concentrations studied, and the SAR changes only marginally from 70 to 60 W/g. From the initial slope of the T vs. t curves shown in Fig. 7a, SAR values were calculated for all four samples and are presented in Fig. 7b. SAR = 110 W/g for MnFe_2_O_4_, 60 W/g for GO-MnFe_2_O_4_, 70 W/g for CoFe_2_O_4_, and 60 W/g for GO-CoFe_2_O_4_. The significant decrease in SAR upon GO incorporation into MnFe_2__2_O_4_ is attributable to the combined effects of: (i) dilution of the magnetic phase by the nonmagnetic GO matrix, reducing the effective M_S_ per gram of composite; (ii) introduction of structural defects including oxygen vacancies and the impurity α-Fe_2_O₃ phase (as identified by XRD), which generate non-isotropic strain and pin magnetic domains, preventing them from responding freely to the AMF; and (iii) interfacial interactions between the ferrite surface and GO functional groups that perturb cation distribution and locally suppress the magnetization, as discussed in the XRD and magnetic sections above. Beyond the anisotropy and relaxation mechanism arguments, the calculated SAR values are also influenced by particle size, morphology, and surface anisotropy. The surface anisotropy contribution, which increases with decreasing particle size due to the growing surface-to-volume ratio, is particularly relevant here. In the framework of Néel’s surface anisotropy model, surface symmetry breaking, arising from structural defects, multifaceted surfaces, broken exchange bonds, coordination number reduction, and surface strain, contributes an additional effective anisotropy term that augments the bulk magnetocrystalline value^89^. The distinct morphologies observed for the two ferrite systems are therefore relevant: MnFe_2_O_4_ exhibits a cubic/cuboid morphology with well-defined facets, while CoFe_2_O_4_ presents a more spherical morphology. Faceted surfaces, as in the cuboid case, offer flat crystallographic planes with relatively ordered surface spin configurations, whereas the nominally spherical CoFe_2_O_4_ particles, which in practice exhibit irregular multi-faceted surfaces at the nanoscale, present more surface disorder^63^. Despite spin canting existing in both systems due to their small particle sizes, the greater surface disorder in the spherical CoFe_2_O_4_ nanoparticles is expected to further enhance the effective surface anisotropy, adding to the already large magnetocrystalline K value and further deepening the Néel relaxation freeze-out in this system. This morphology-dependent surface anisotropy effect thus reinforces the conclusion that CoFe_2_O_4_ operates exclusively in the Brownian relaxation regime under the field and frequency conditions of this study. Meanwhile, MnFe_2_O_4_ benefits from a more thermally accessible combined relaxation mechanism that underpins its superior heating performance. Note: The numerical estimates for τ_N_ and τ_B_ are derived from the particle sizes and anisotropy constants stated in the manuscript.

By comparing our results to literature (Table 1) and (Table 2), we found that SAR values reported in this work are higher than or comparable to many of the published works under same conditions of particle size, magnetization and AMF parameters. Samples showing higher SAR values are either caped with some biocompatible materials, having larger particle size, and/or AMF parameters like field strength and frequency are higher than those values adopted in the current work, 300 Oe and 304 kHz.

**Table 1:** Comparison of our data with the list of published similar work of MnFe_2_O_4_.

No.	Sample	M_s_(emu/g)	SAR (W/g)	f (kHz)	H (Oe)	D (nm)	Ref.
1	MnFe_2_O_4_	67	356.17	265	335.2	13	^90^
2	MnFe_2_O_4_	–	295	195	628.3	20	^91^
3	MnFe_2_O_4_	58.8	148.4	1950	56.54	19	^44^
4	MnFe_2_O_4_/SiO_2_	-	336	400	320	18	^92^
5	PVP/MnFe_2_O_4_	60	468.37	265	335.2	13	^93^
6	Mn@silica	40	84.65	260	69.11	14	^94^
8	MnFe_2_O_4_@Chitosan	-	320	342	200	15	^95^
9	MnFe_2_O_4_	56.4	145.78	316	295	9	^96^
10	MnFe_2_O_4_ GO/MnFe_2_O_4_	50 10	90	310	300	17	^97^
			170	310	700		
			80	310	300		
			280	310	800		
12	MnFe_2_O_4_ GO/ MnFe_2_O_4_	60 50	110	304	300	14.89 15.5	This work
			60	304	300		

**Table 2:** Comparison of our data with the list of published similar work of CoFe_2_O_4_.

No.	Sample	MS (emu/g)	SAR (W/g)	Freq. (kHz)	Field (Oe)	D (nm)	Ref.
1	GO/ CoFe_2_O_4_	40	2 mg 77.4 1 mg 232.5	200 150	300	5	^98^
2	CoFe_2_O_4_@OA/OLA	70	297	450	300	6–20	^99^
3	Aminodextran/ CoFe_2_O_4_	50	259 518	327 981	170 230	10–50	^100^
4	CoFe_2_O_4_	63	133	101	510	28	^101^
5	CA- CoFe_2_O_4_	43	76	329	1201	4 to 7	^102^
6	CoFe_2_O_4_	55.3	9.6	85	80	7.2	^103^
7	CoFe_2_O_4_	63	29	300	188	6	^104^
8	CoFe_2_O_4_	60	99	420	187	8.9	^105^
9	CoFe_2_O_4_	71	185.32	316	295	10	^106^
10	CoFe_2_O_4_	80	248	350	440	9.45	^107^
11	CoFe_2_O_4_	39	142	178	80	13	^108^
12	GO/CoFe_2_O_4_ CoFe_2_O_4_	40 35	70 60	304 304	300 300	12.5 14	This work

## Conclusion

This study presents a systematic investigation of MnFe_2_O_4_ and CoFe_2_O_4_ nanoparticles and their graphene oxide composites synthesized via a hydrothermal approach, with the aim of elucidating the relationship between magnetic anisotropy, relaxation dynamics, and hyperthermia performance. The central finding is that magnetic anisotropy is the primary determinant of heating efficiency under the experimental AMF conditions (300 Oe, 304 kHz): the low-anisotropy MnFe_2_O_4_ system (K = 3.3 × 10^3^ J/m^3^) benefits from concurrent Néel and Brownian relaxation mechanisms whose effective relaxation time is better matched to the applied frequency, yielding a SAR of 110 W/g. In contrast, the high-anisotropy CoFe_2_O_4_ system (K = 2.7 × 10^5^ J/m^3^) operates exclusively through Brownian relaxation, as its Néel relaxation is thermally inaccessible under these conditions, resulting in a substantially lower SAR of 70 W/g. These results demonstrate that maximizing SAR in ferrite-based hyperthermia agents requires not only high saturation magnetization but careful tuning of the anisotropy constant to match the relaxation timescale to the applied field frequency, a design principle that is clearly illustrated by the contrasting behavior of the soft and hard ferrite systems studied here. The incorporation of graphene oxide introduced competing effects: while it improved colloidal dispersion and offers a surface platform amenable to bioconjugation and functionalization for targeted drug delivery, it consistently reduced SAR values across both ferrite systems. In MnFe_2_O_4_-GO, the SAR reduction from 110 to 60 W/g is attributed to magnetic phase dilution by the nonmagnetic GO matrix, domain pinning by interfacial strain and structural defects, and the suppression of hematite impurity phases that, while removing a non-contributing secondary phase, also reflects broader perturbations to the magnetic microstructure at the ferrite–GO interface. In CoFe_2_O_4_-GO, the marginal SAR change from 70 to 60 W/g is consistent with the Brownian-dominated mechanism in this system being relatively insensitive to the internal magnetic modifications introduced by GO. Taken together, these findings establish that the trade-off between the dispersibility benefits and the SAR penalties of GO incorporation is system-dependent and must be evaluated in the context of the dominant relaxation mechanism. From a materials design perspective, this work identifies MnFe_2_O_4_ as the more promising candidate for magnetic hyperthermia among the systems studied, owing to its favorable combination of magnetization, anisotropy, and relaxation dynamics at clinically relevant field conditions. The particle size (~ 23 nm), morphology, and surface anisotropy contributions further modulate its performance and offer clear handles for optimization. Future work should focus on systematic variation of particle size and GO loading to map the full SAR parameter space, surface functionalization of the composites with targeting ligands for tumor-specific delivery, and evaluation of heating performance in viscous and gel-like media that more closely approximate the intratumoral environment, where Brownian relaxation is suppressed and Néel relaxation becomes the exclusive heating pathway, a condition under which the anisotropy contrast between MnFe_2_O_4_ and CoFe_2_O_4_ would be expected to become even more pronounced. Ultimately, in vitro cytotoxicity assays and in vivo hyperthermia efficacy studies are essential next steps toward translating these findings into clinically viable therapeutic platforms.

## Data Availability

The datasets generated and/or analysed during the current study are available from the corresponding author on reasonable request.
